# Multimodal imaging in sclerochoroidal calcification: a case report and literature review

**DOI:** 10.1186/s12886-020-01520-y

**Published:** 2020-06-22

**Authors:** Mizuho Mitamura, Satoru Kase, Susumu Ishida

**Affiliations:** 1 Department of Ophthalmology, Faculty of Medicine and Graduate School of Medicine, Hokkaido University, N-15, W-7, Kita-ku, Sapporo, 060-8638 Japan; 2 Department of Ophthalmology, Teine Keijinkai Hospital, Sapporo, Japan

**Keywords:** Sclerochoroidal calcification, Chronic kidney disease, Indocyanine green angiography, Laser speckle flowgraphy

## Abstract

**Background:**

Sclerochoroidal calcification (SCC), a rare condition found in elderly people, is idiopathic or occasionally secondary to disorders affecting calcium metabolism. Findings of multimodal imaging including choroidal circulation are, however, largely unknown. We present a patient of SCC with systemic background, who underwent multimodal imaging evaluations.

**Case presentation:**

A 70-year-old Japanese man was referred to our clinic because of bilateral fundus lesions. He had a history of chronic kidney disease (CKD) and secondary hyperparathyroidism. Fundus photography showed a cluster of choroidal folds in the superotemporal extra-macular region OS. Swept-source optical coherence tomography demonstrated ellipsoid zone disruption OD, retinal pigment epithelium undulation OS, dilated Haller layer veins OU, and central choroidal thickening OU and thinning of the overlying choroid due to scleral elevation OS. Fluorescein angiography detected macular hyperfluorescence OD. Indocyanine green angiography demonstrated choroidal vascular hyperpermeability together with numerous scattered hypofluorescent lesions OU. Fundus autofluorescence showed multiple hypoautofluorescent spots surrounded by hyperautofluorescent areas OD. Laser speckle flowgraphy exhibited choroidal blood flow reduction represented by a cold color pattern OU. B-mode echography displayed hyperechoic solid lesions with acoustic shadowing and orbital computed tomography revealed high density areas in the sclera, both of which were consistent with calcification. The patient was diagnosed with SCC, and these imaging findings remained unchanged 7 months after the diagnosis.

**Conclusions:**

We reported a case of SCC with the background of CKD. Our detailed multimodal observations indicated choroidal hypoperfusion possibly caused by mechanical compression due to calcium deposition in the sclera.

## Background

Sclerochoroidal calcification (SCC) is a rare condition that can be incidentally identified as abnormal fundus lesions in the elderly during routine ophthalmological examinations [1]. In addition to its idiopathic etiology, SCC can be associated with conditions affecting calcium metabolism such as hyperparathyroidism. Shields et al. reported a case series of SCC, the cause and frequency of which were hyperparathyroidism (27%) with parathyroid adenoma (15%), Gitelman syndrome (11%), and Bartter syndrome (2%) [2]. SCC is characterized by yellow-whitish irregular subretinal lesions commonly localized in the superotemporal extra-macular to mid-periphery of the fundus, around the postequatorial regions, and along the temporal vascular arcades, and typically asymptomatic with no exudative lesions [3]. Despite its nomenclature, calcium deposition was shown to be confined to the sclera, but not to the choroid, by enhanced depth imaging optical coherence tomography (EDI-OCT) observations [4]. However, little is known about choroidal circulation changes in SCC. We herein present a case of SCC with the background of chronic kidney disease (CKD) and review the literature on multimodal imaging analyses.

## Case presentation

A 70-year-old Japanese man complained of blurred vision in both eyes and was referred to our clinic because of bilateral fundus lesions. He had a history of CKD and secondary hyperparathyroidism with no family history. CKD is defined as any renal disorder lasting more than 3 months, the severity of which is classified into 1 to 5 stages based on its etiology, urine protein levels and renal function. This patient suffered stage 5 CKD (i.e., end-stage renal failure) of unknown etiology, having received hemodialysis for 35 years.

**Fig. 1:**
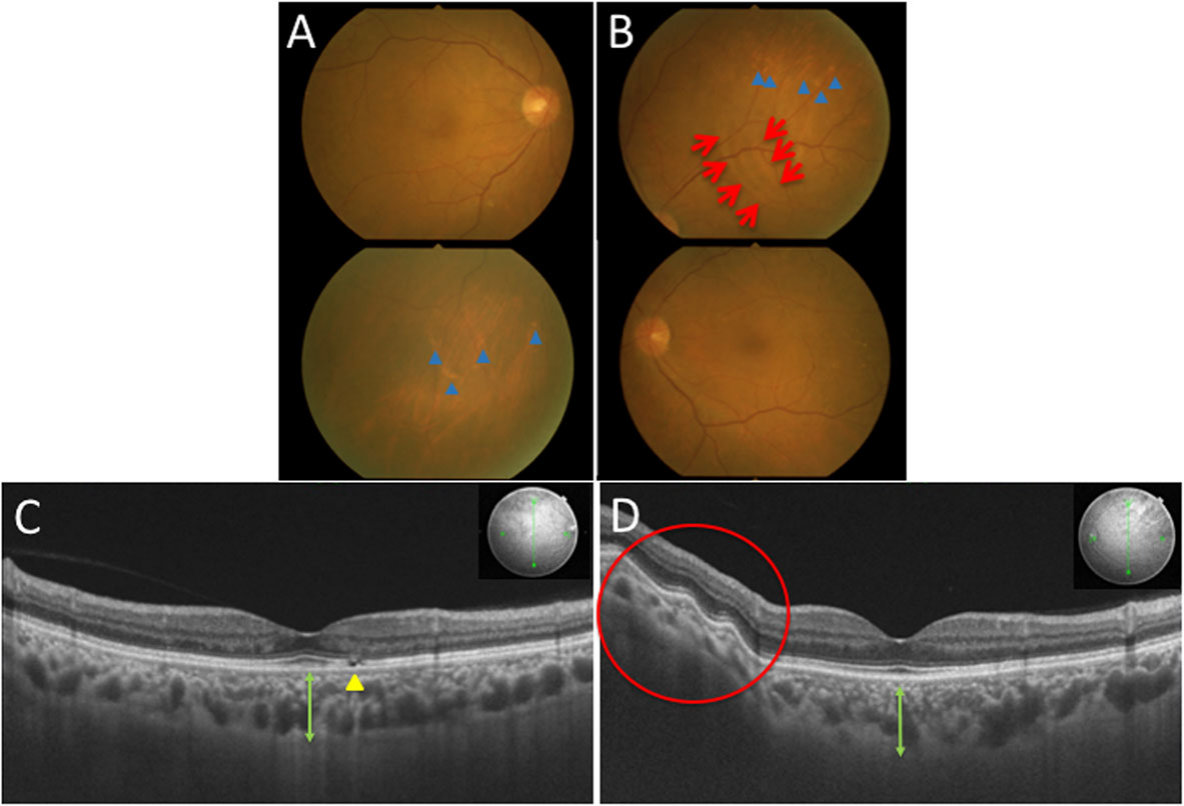
Initial findings on color fundus photography (CFP) and swept-source optical coherence tomography (SS-OCT) in the present case with sclerochoroidal calcification (SCC) secondary to chronic kidney disease (CKD). **a** CFP in the right eye showing pale choroidal lesions in the inferior mid-periphery (blue arrowheads). **b** CFP in the left eye showing a cluster of choroidal folds in the superotemporal extra-macular region (red arrows) and pale choroidal lesions in the superior regions (blue arrowheads). **c** SS-OCT in the right eye at vertical scans through the central fovea showing ellipsoid zone (EZ) disruption (yellow arrowhead) and dilated Haller layer veins, together with central choroidal thickness exceeding 300 μm (green arrow). **d** SS-OCT in the left eye at vertical scans through the central fovea showing retinal pigment epithelium (RPE) undulation at the site of choroidal thinning due to scleral elevation (red circle). Dilated Haller layer veins were prominent together with central choroidal thickness exceeding 300 μm (green arrow)

**Fig. 2:**
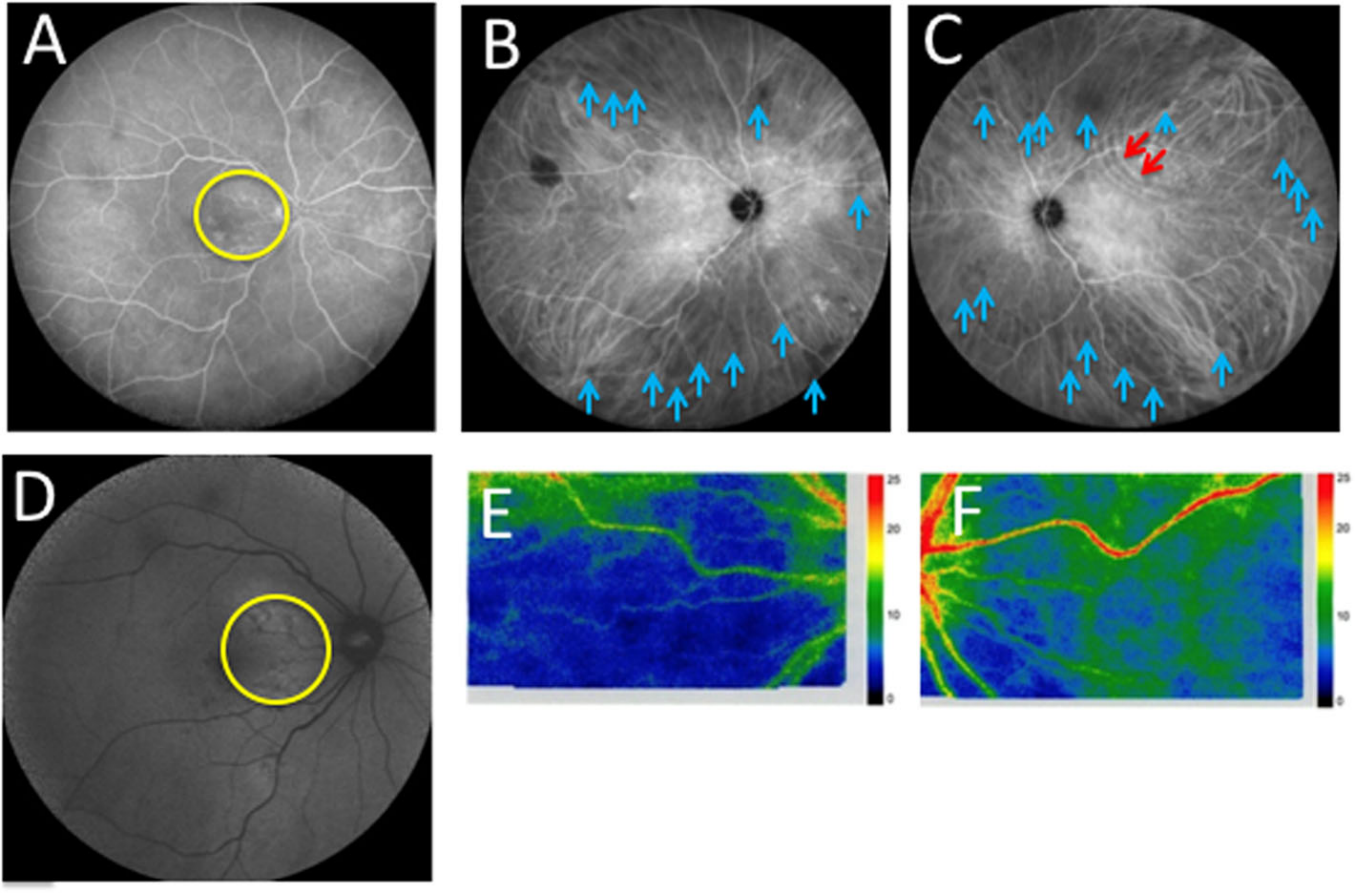
Initial findings on fluorescein angiography (FA), indocyanine green angiography (ICGA), fundus autofluorescence (FAF), and laser speckle flowgraphy (LSFG) in the present case with SCC secondary to CKD. **a** FA showing hyperfluorescence (yellow circle) in the right eye. **b**, **c** ICGA showing choroidal vascular hyperpermeability (CVH) at the macular area together with numerous scattered hypofluorescent lesions (blue arrows) in both eyes and linear hyperfluorescent lesions (red arrows) in the left eye (**c**). **d** FAF in the right eye showing multiple hypoautofluorescent spots surrounded by hyperautofluorescent areas (yellow circle). **e**, **f** LSFG showing choroidal blood flow reduction represented by a colder color pattern in the right eye (**e**) than in the left eye (**f**)

**Fig. 3:**
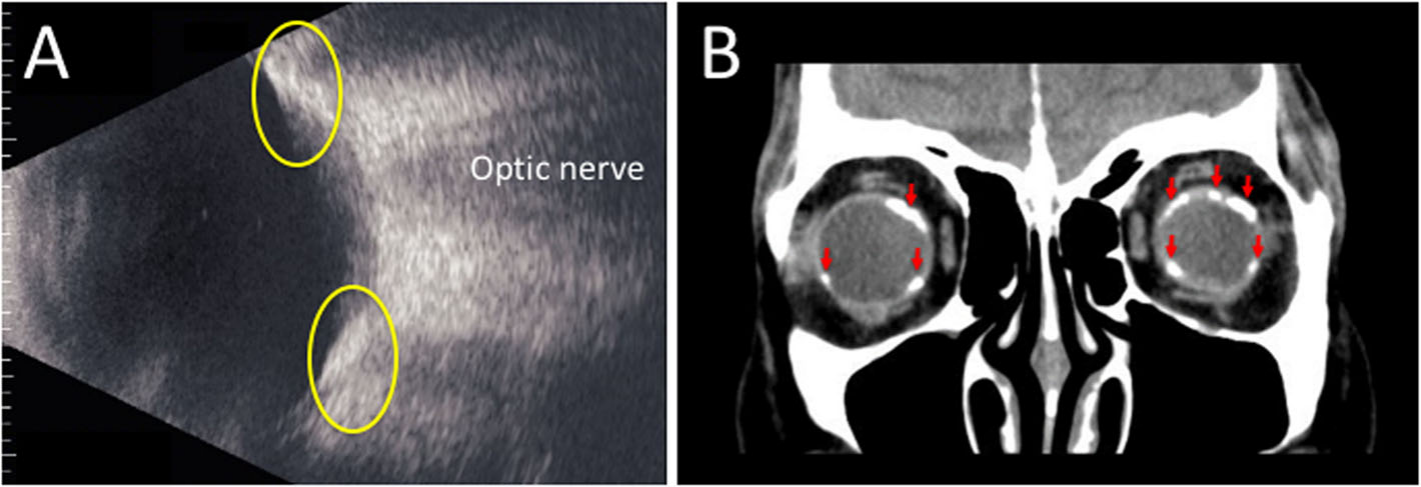
Initial findings on B-mode echography and orbital computed tomography (CT) in the present case with SCC secondary to CKD. **a** B-mode echography in the left eye at a vertical scan through the optic nerve showing hyperechoic lesions with acoustic shadowing (yellow circles). **b** Orbital CT at a coronal scan through the eye globes showing scleral high-density areas (red arrows)

His best-corrected visual acuity was 0.9 OD and 1.0 OS with mild myopia, and his intraocular pressure was normal OU. Slit-lamp microscopy did not detect any findings except mild cataract OU. Color fundus photography (CFP) revealed pale choroidal lesions in the inferior mid-periphery OD and in the superior and inferonasal regions OS (Fig. 1a and b, blue arrowheads). A cluster of choroidal folds in the superotemporal extra-macular region OS (Fig. 1b, red arrows) and a choroidal nevus in the temporal mid-periphery OD were also observed. Swept-source OCT (SS-OCT) demonstrated parafoveal ellipsoid zone (EZ) disruption OD (Fig. 1c, yellow arrowhead), retinal pigment epithelium (RPE) undulation OS (Fig. 1d, red circle) and dilated Haller layer veins OU. Choroidal thickness was more than 300 μm OU at the central fovea (Fig. 1c and d, green arrows) and attenuated by scleral elevation around the upper arcade (Fig. 1d, red circle). Fluorescein angiography (FA) detected hyperfluorescent spots at the macula from the early to late phases OD (Fig. 2a, yellow circle), whereas no change was found OS. Indocyanine green angiography (ICGA) demonstrated choroidal vascular hyperpermeability (CVH) at the macular areas OU, together with numerous scattered hypofluorescent lesions in the mid-venous phase OU (Fig. 2b and c, blue arrows). Linear hyperfluorescent lesions were detected OS (Fig. 2c, red arrows) corresponding to the extra-macular choroidal folds on CFP and SS-OCT. Fundus autofluorescence (FAF) showed multiple hypoautofluorescent spots surrounded by hyperautofluorescent areas at the posterior pole OD, suggesting RPE disruption (Fig. 2d, yellow circle), whereas no significant abnormalities were found OS. Laser speckle flowgraphy (LSFG) is a blood flow imaging device based on laser scattering, which non-invasively allows for two-dimensional visualization of fundus circulation in diseases such as intraocular tumor [5]. On LSFG, both eyes exhibited macular hypoperfusion represented by cold colors, and the right eye with RPE/EZ disruption at the macula showed a colder color pattern than the left eye (Fig. 2e and f). B-mode echography displayed hyperechoic solid lesions with acoustic shadowing (Fig. 3a, yellow circles), which indicated calcification in concert with bilateral scleral high-density areas on orbital computed tomography (Fig. 3b, red arrows).

Laboratory tests revealed that serum creatinine levels increased at 6.35 mg/dl. Serum calcium and phosphorus levels slightly decreased to 8.3 mg/dl (normal: 8.8–10.1), and 2.6 mg/dl (normal: 2.7–4.6), respectively. Vitamin D3 levels decreased to 9 pg/ml (normal: 20–60). Since he had taken a daily oral use of the vitamin D analogue alfacalcidol, parathyroid hormone levels were within the normal range. The patient was diagnosed with SCC based on the multimodal imaging characteristics. The patient’s ocular manifestation was observed with no treatment. His ophthalmological findings remained unchanged 7 months after the diagnosis.

## Discussion and conclusion

The present study demonstrated novel findings on SCC via multimodal imaging techniques, so as to better understand clinical manifestations of the disease. To the best of our knowledge, this report is the first to show choroidal blood flow impairment in SCC. As seen in the current case, outer retinal abnormalities [4], RPE disruption [6], and CVH [7] were previously noted and these lesions existed within the extent of inwardly protruding sclera (i.e., calcified mass), suggesting the involvement of direct compression to the overlying tissue. In the current case, however, these lesions were observed in the macular area free of SCC, highlighting the potential impact of other accompanying mechanisms rather than direct compression.

Fung et al. analyzed 9 cases of SCC using EDI-OCT, and demonstrated that calcium deposition developed exclusively within the sclera exerting compression to the overlying choroid. They referred to the anterior surface of the calcified sclera as “rocky” and “rolling” appearances [4]. The rocky contour showed abrupt elevation, often with a pointed surface leading to outer retinal destruction such as absence of the external limiting membrane and EZ. The rolling contour showed more gentle elevation with a smooth surface undulation. In our case, SS-OCT showed mild scleral elevation with choroidal folds but little or no damage to the overlying retinal tissue (Fig. 1d, oblique tissue structure not imaged due to the nature of this scanning method). The surface configuration of this condition would therefore be consistent with the rolling appearance.

Hyperautofluorescent findings on FAF are typically related to increased lipofuscin accumulation in metabolically stressed or diseased RPE. Along with progressive RPE dysfunction, hyperautofluorecence then turns to hypoautofluorescence indicative of RPE atrophy. Caminal-Mitjana et al. compared FAF and spectral-domain OCT findings in 3 cases of SCC and revealed a mixture of hyper- and hypoautofluorescence depending on the degree of compression [6]. They hypothesized the growing mass effect of SCC exerting chronic compression to the choriocapillaris and thus compromising oxygen supply to RPE and the outer retina. Indeed, hyperautofluorescence was associated with choriocapillaris attenuation, whereas hypoautofluorescence was observed at the area of elevated RPE [6]. In our case, FAF showed a mixture of hyper- and hypoautofluorescence at the macular area of the right eye; however, the RPE changes could not be attributed to direct compression given the absence of a calcified mass beneath the lesion.

There were few reports describing ICGA findings in patients with SCC. In a previous case report, ICGA showed hyperfluorescence, which appeared to be CVH, in the corresponding region of SCC [7]. In our patient, ICGA detected CVH at the macular area free of SCC, suggesting an alternative etiology other than direct compression. Importantly, ICGA also showed numerous scattered hypofluorescent lesions, suggesting circulation impairment in the entire choroid. In this case, CVH and choroidal hypoperfusion would theoretically be explained by the etiology of broad choroidal congestion secondary to multiple sites of compression from the calcified sclera. This led reasonably to the loss of oxygen supply to the outer retina and RPE at the macula, in consistence with the cold color pattern on LSFG. Moreover, our patient received hemodialysis due to CKD, suggesting the possible involvement of systemic vascular dysfunction such as overhydration and hypotension, both of which may have resulted in CVH and choroidal hypoperfusion, respectively.

In conclusion, we reported a case of SCC with the background of CKD. Our detailed multimodal imaging analyses for the first time demonstrated choroidal circulation disturbance possibly caused by mechanical compression due to massive calcium deposition in the sclera. Further investigation into SCC patients without CKD would be needed in the future to validate our current hypothesis.

## Data Availability

N/A

## Abbreviations

SCC: Sclerochoroidal calcification
EDI-OCT: Enhanced depth imaging optical coherence tomography
CKD: Chronic kidney disease
CFP: Color fundus photography
EZ: Ellipsoid zone
SS-OCT: Swept source optical coherence tomography
RPE: Retinal pigment epithelium
FA: Fluorescein angiography
ICGA: Indocyanine green angiography
CVH: Choroidal vascular hyperpermeability
FAF: Fundus autofluorescence
LSFG: Laser speckle flowgraphy
CT: Computed tomography
